# Survival of hypoxia-induced dormancy is not a common feature of all strains of the *Mycobacterium tuberculosis* complex

**DOI:** 10.1038/s41598-021-81223-6

**Published:** 2021-01-29

**Authors:** Barbara Tizzano, Tobias K. Dallenga, Christian Utpatel, Jochen Behrends, Susanne Homolka, Thomas A. Kohl, Stefan Niemann

**Affiliations:** 1 Molecular and Experimental Mycobacteriology, Research Center Borstel, Leibniz Lung Center, Parkallee 1-40, 23845 Borstel, Germany; 2 Cellular Microbiology, Research Center Borstel, Leibniz Lung Center, Parkallee 1-40, 23845 Borstel, Germany; 3 Core Facility Fluorescence Cytometry, Research Center Borstel, Leibniz Lung Center, Parkallee 1-40, 23845 Borstel, Germany; 4 German Center for Infection Research, Borstel Site, Borstel, Germany

**Keywords:** Bacteria, Pathogens, Tuberculosis

## Abstract

While persistence in a dormant state is crucial for the life cycle of *Mycobacterium tuberculosis*, no investigation regarding dormancy survival of different strains across different lineages was performed so far. We analyzed responses to oxygen starvation and recovery in terms of growth, metabolism, and transcription. All different strains belonging to the Euro-American lineage (L4) showed similar survival and resuscitation characteristics. Different clinical isolates from the Beijing (L2), East African-Indian (L3), and Delhi/Central Asian (L1) lineage did not survive oxygen starvation. We show that dormancy survival is lineage-dependent. Recovery from O_2_ starvation was only observed in strains belonging to the Euro-American (L4) lineage but not in strains belonging to different lineages (L1, L2, L3). Thus, resuscitation from dormancy after oxygen starvation is not a general feature of all *M. tuberculosis* strains as thought before. Our findings are of key importance to understand infection dynamics of non-Euro-American vs Euro-American strains and to develop drugs targeting the dormant state.

## Introduction

Tuberculosis (TB) is caused by bacteria belonging to the *Mycobacterium tuberculosis* complex (MTBC), a panel of organisms with considerable genomic diversity and a phylogeographical population structure^1,2^. On a global level, MTBC strains can be classified into eight main lineages: Indo-Oceanic, including East African Indian (EAI) strains (L1); East-Asian, including Beijing (L2); Delhi/Central Asian (Delhi/CAS) (L3); Euro-American (L4); West African 1 (L5); West African 2 (L6); Ethiopia (L7), and a lineage that seems to be restricted to the African Great Lakes region (L8)^3^. These lineages can be further distinguished in several sub-lineages, e.g. within L4^4–7^, or clustered in ancestral *M. tuberculosis* (L1, L7–8), modern *M. tuberculosis* (L2–4), and *M. africanum* (L5–6). Modern lineages are defined by lacking the *M. tuberculosis*-specific deletion 1 region (TbD1) genomic segment^8^. Moreover, the MTBC includes an “animal lineage” that comprises mycobacteria that cause disease in different animals, e.g. *M. bovis*^9^. Strains of different lineages show significant diversity on genomic level that translates into differences in their pathobiology, e.g. pathogenesis, induced immune responses, disease outcome, and vaccine efficacy^4,10–12^. For instance, Beijing (L2) and Euro-American Haarlem (L4) strains have been described to exhibit more virulent phenotypes compared to the ones that belong to the East African-Indian group (L1)^12^. While genomic differences between lineages have been reported in various studies, unveiling the precise mechanisms behind differences in pathogenesis and virulence remains a big challenge.

One of the hallmarks in mycobacterial pathogenesis is overcoming host immune defense after infection and surviving in a dormant state, thus, causing a latent, asymptomatic infection that may last for years^13,14^. Mycobacterial dormancy is thought to be induced as a response to stresses imposed by the immune system. In contrast, persistent *M. tuberculosis* are rather defined as a non-growing population prior to any stress that is able to randomly switch between replicating and non-replicating phenotypes independent of environmental stresses^15^. Classically, dormancy has been examined by using a hypoxia model^16^, which was also exclusively used in this study. However, a dormant state of *M. tuberculosis* can be induced by almost any kind of induced stress, e.g. any nutrient depletion^14,17^. Latent tuberculosis infection has been associated with containment of the pathogen within a complex structure denoted as granuloma: a focal collection of inflammatory cells surrounding bacteria in a dynamic process of continual immunologic control of replication and prevention of dissemination^14,18^. The physiological environment of granulomas has been described to be hypoxic^19^ and acidic^14^. MTBC strains are sensitive to the level of oxygen present and respond to oxygen availability by changes in growth, metabolic activity and transcriptional profile^18^. It has been demonstrated that gradual depletion of oxygen causes a shift-down of aerobically growing mycobacteria to an anaerobic synchronized state of dormancy. Lawrence Wayne has conducted pioneering dormancy studies of *M. tuberculosis*^20^. Wayne and colleagues showed in vitro that gradual, self-generated oxygen depletion triggers a dormancy response in *M. tuberculosis* similar to the one found in granulomas^16,20^. According to this observation, the bacterial culture, incubated in sealed tubes with a defined headspace, slowly adapt and survive anaerobiosis by shifting down to a non-replicating, dormant state^21^. The shift-down to dormancy can be divided into three stages^16^. The first phase, occurring after the initial aerobic exponential growth, was termed non-replicating persistence stage 1 (NRP-1). It is characterized by slower increase in culture medium turbidity, oxygen levels of 10% and increased antibiotic tolerance. After NRP-1, the bacteria enter a phase where no further increase in culture medium turbidity is seen and the cells are arrested at a uniform, synchronized stage in cell cycle (NRP-2). The last phase occurring in H37Rv (L4) after 3–4 weeks is the dormancy phase, a viable but non-cultivable state (VBNC). This phase is characterized by a shutdown of the main replicative and metabolic activities. Such dormant bacterial cultures can be distinguished from dead ones, because they regain metabolic activity and the ability to grow and divide after reintroduction of oxygen.

For several years, the phenomenon of non-cultivability of bacterial cells has been a matter of intensive debate and different modifications of Wayne conditions have been assayed to assess hypoxia-induced dormancy in vitro^20^. In such models, bacterial cultures ultimately lose the ability to form colonies on solid media, but they can resuscitate in liquid media either spontaneously or upon addition of proteinaceous reactivation factors^22^. However, there is consensus that there is no resuscitation (meant as a restoration of normal metabolic function and growth) without a reactivating trigger^23^. Importantly, most experiments addressing hypoxia-induced dormancy adaptation and in vitro reactivation have been performed with the laboratory strain H37Rv (L4). Data on more diverse collections of clinical isolates are only sparsely available^24^. Accordingly, general knowledge on hypoxia-induced dormancy and reactivation is virtually built on studies performed on one single strain that has been used in the laboratory for decades. As the dormancy state induced by hypoxia has been considered as highly relevant for vaccine as well as drug development, this represents a significant gap of knowledge, especially as studies have confirmed a higher genomic diversity in clinical MTBC isolates as previously anticipated^1,4,5^.

To address this question, we investigated a panel of MTBC strains representing major phylogenetic lineages of the MTBC in the Wayne dormancy model. Our findings show that not all clinical isolates survived prolonged periods of oxygen starvation. In fact, only those isolates that belong to Euro-American (L4) survived hypoxia-induced dormancy, while others, EAI (L1), Beijing (L2), and Delhi/CAS (L3), did not. This observation may help to better understand the balance between hypoxia-induced dormancy and reactivation in TB.

## Results

### Hypoxic conditions result in dormancy as characterized by growth arrest and loss of metabolic activity

The major aim of our study was to investigate hypoxia-induced dormancy survival and resuscitation of clinical strains of the Euro-American (L4) and Beijing (L2) lineages, both being reported to be highly successful on a global level with regard to spread and virulence^25^. We used the Wayne model as previously described^16^ and made similar observations as originally published (Supplementary Figs. S1, S2).

**Figure 1:**
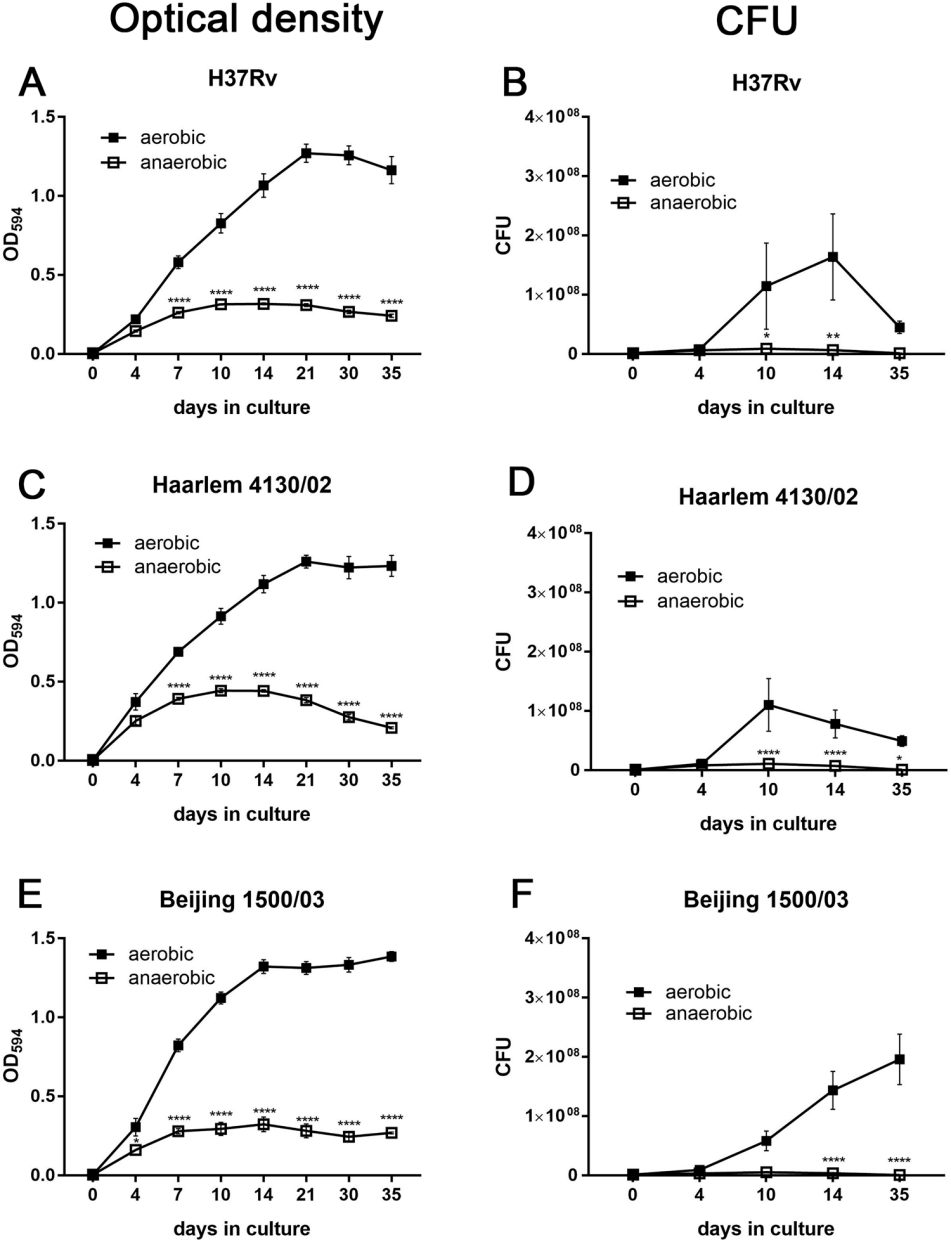
O_2_ depletion-induced dormancy in H37Rv (L4), Haarlem (L4) and Beijing (L2). Approximately 2 × 10^6^ (OD = 0.004) mycobacteria were cultured with or without O_2_. At indicated time points, OD was measured (**A**,**C**,**E**) or bacteria were plated for CFU analysis (**B**,**D**,**F**). O_2_ depletion led to reduced bacterial growth in all examined strains (H37Rv, Haarlem, Beijing). n = 4 (H37Rv, Beijing), n = 3 (Haarlem) independent experiments with triplicates in each, ****p < 0.0001, **p < 0.005, *p < 0.05, two-way ANOVA.

We tested H37Rv (L4) (ATCC27294, internal identification number 9679/00) and a Haarlem (L4) strain (4130/02) as representatives of the Euro-American lineage and a Beijing (L2) strain (1500/03) for hypoxia-induced dormancy analyses. An overview of the MTBC population structure and phylogenetic lineages of the strains used in this study is depicted in Supplementary Fig. S3. Oxygen depletion within 2–3 weeks was visualized by Methylene Blue fading (Supplementary Fig. S1). All three strains (H37Rv (L4) 9679/00, Haarlem (L4) 4130/02, and Beijing (L2) 1500/03) showed similar growth profiles with a reduced growth rate observed for cultures under anaerobic conditions and a growth arrest after 10–14 days (Fig. 1). Additionally, also other clinical isolates of Haarlem (L4) (2336/02) and Beijing (L2) (49/02, 12,594/02) acquired dormant state after oxygen starvation (Supplementary Fig. S4), suggesting that these observations are not specific for particular clinical isolates.

**Figure 2:**
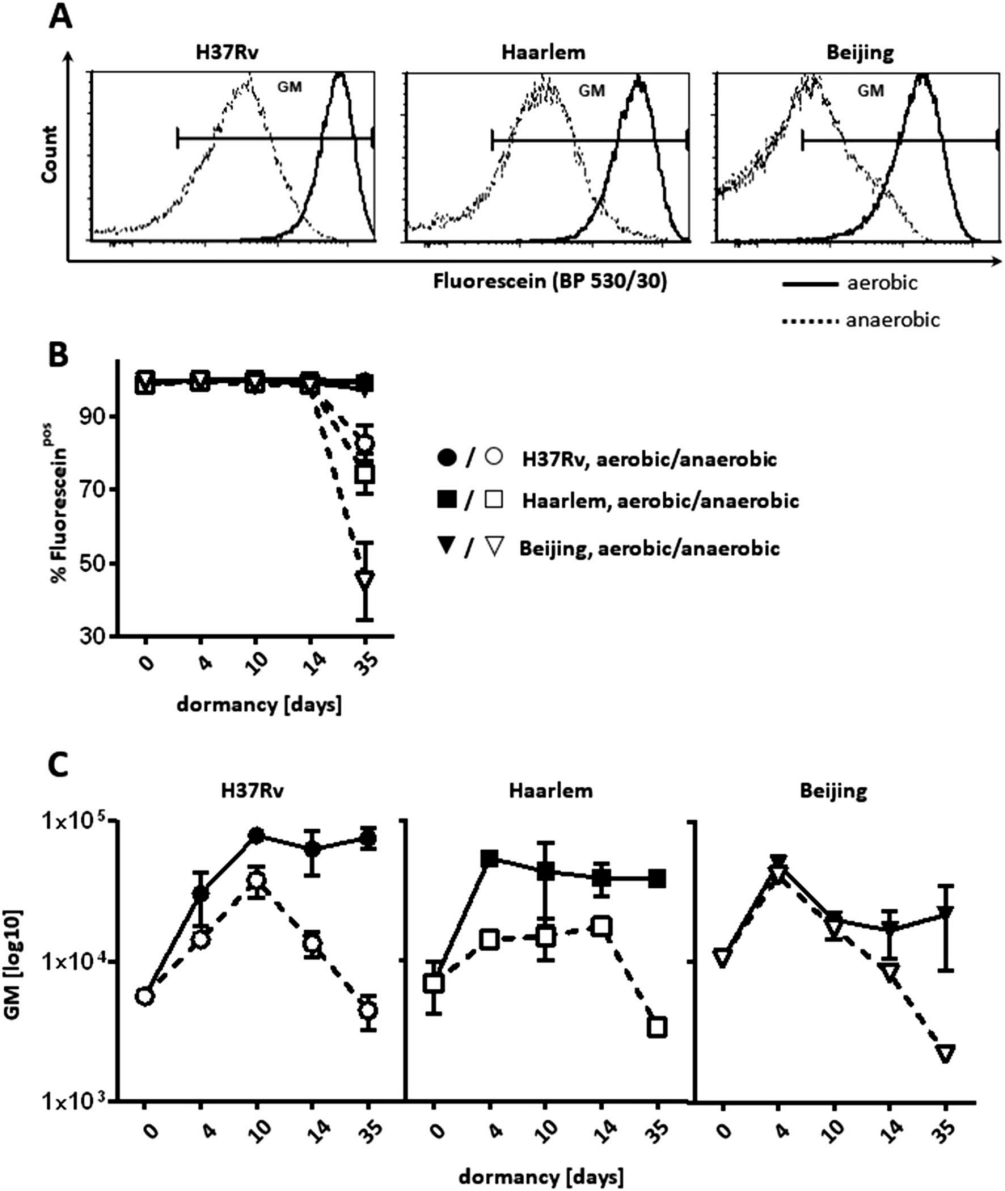
Gradual loss of metabolic activity during dormancy (d0–d35). Example of a histogram of fluorescin-positive mycobacteria at d35 of dormancy after incubation at 37 °C with FDA for 20 min (**A**). Per cent of fluorescein-positive cells (**B**) and geometric mean (GM) fluorescence intensity (**C**) of mycobacterial strains within gate GM were measured at indicated time points. One representative experiment (triplicates) out of two is shown.

In addition to growth and viability, monitored by OD_594_ measurement and CFU, we assessed loss of metabolic activity during oxygen starvation by hydrolysis of fluorescein diacetate (FDA) via flow cytometry. Only viable mycobacteria hydrolyze FDA to free fluorescein by active esterases. The shift-down to dormancy and the decrease in growth rates under hypoxic conditions corresponded with the loss of metabolic activity and subsequent decrease in fluorescence (Fig. 2). H37Rv (L4), Haarlem (L4) and Beijing (L2) strains showed a strong decrease in esterase activity 35 days after oxygen starvation when compared to non-starved cultures as indicated by percentages of fluorescein-positive mycobacteria (Fig. 2A,B). Mean fluorescence intensities of free fluorescein were lowest after 35 days of oxygen depletion (Fig. 2A,C). Thus, all strains examined entered a dormant state at least after 10–14 days of oxygen starvation.

### H37Rv (L4) and Haarlem (L4) but not Beijing (L2) strains reactivated upon oxygen exposure after hypoxia-induced dormancy

**Figure 3:**
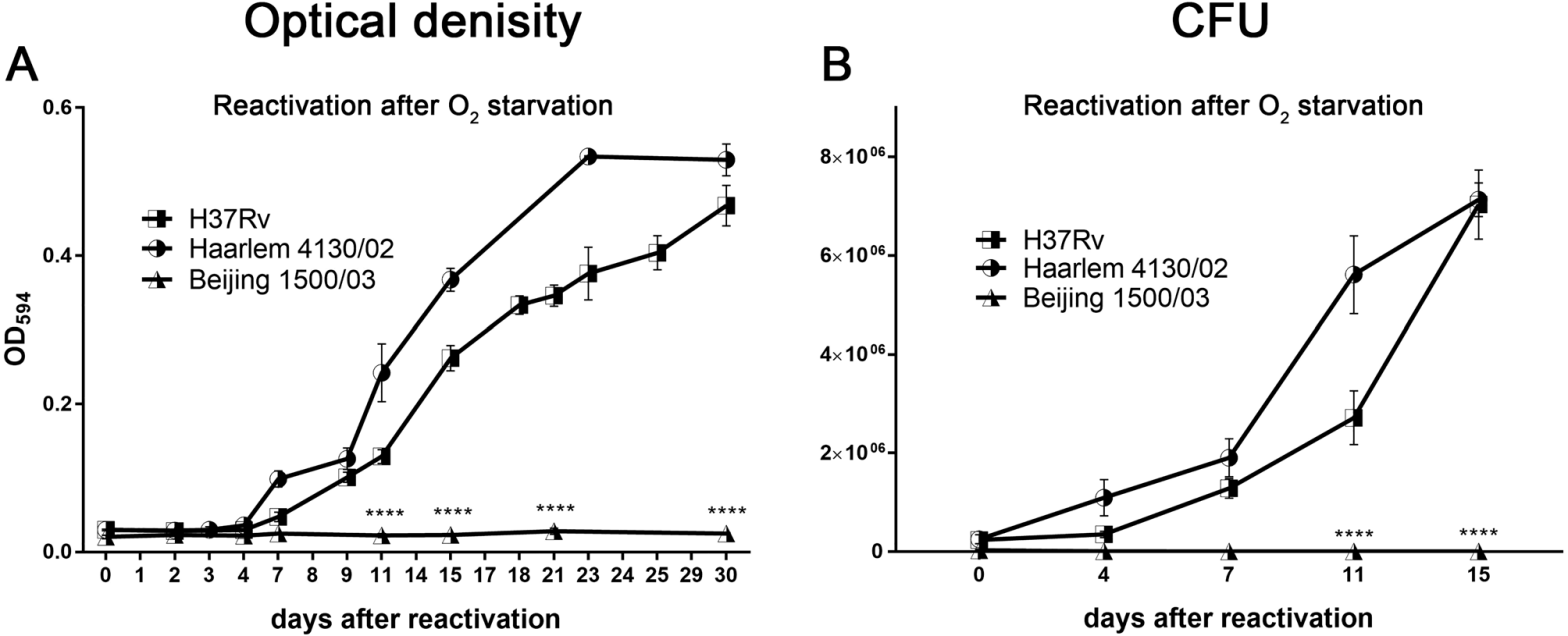
After hypoxia-induced dormancy, H37Rv (L4) and Haarlem (L4), but not Beijing (L2) reactivated by oxygen exposure. 35 days after oxygen starvation, *M. tuberculosis* strains were inoculated in fresh medium under atmospheric oxygen conditions. At indicated time points, mycobacterial growth was measured by optical density (**A**) or bacteria were plated for CFU analysis (**B**). n = 3 independent experiments with triplicates in each, ****p < 0.0001, two-way ANOVA.

**Figure 4:**
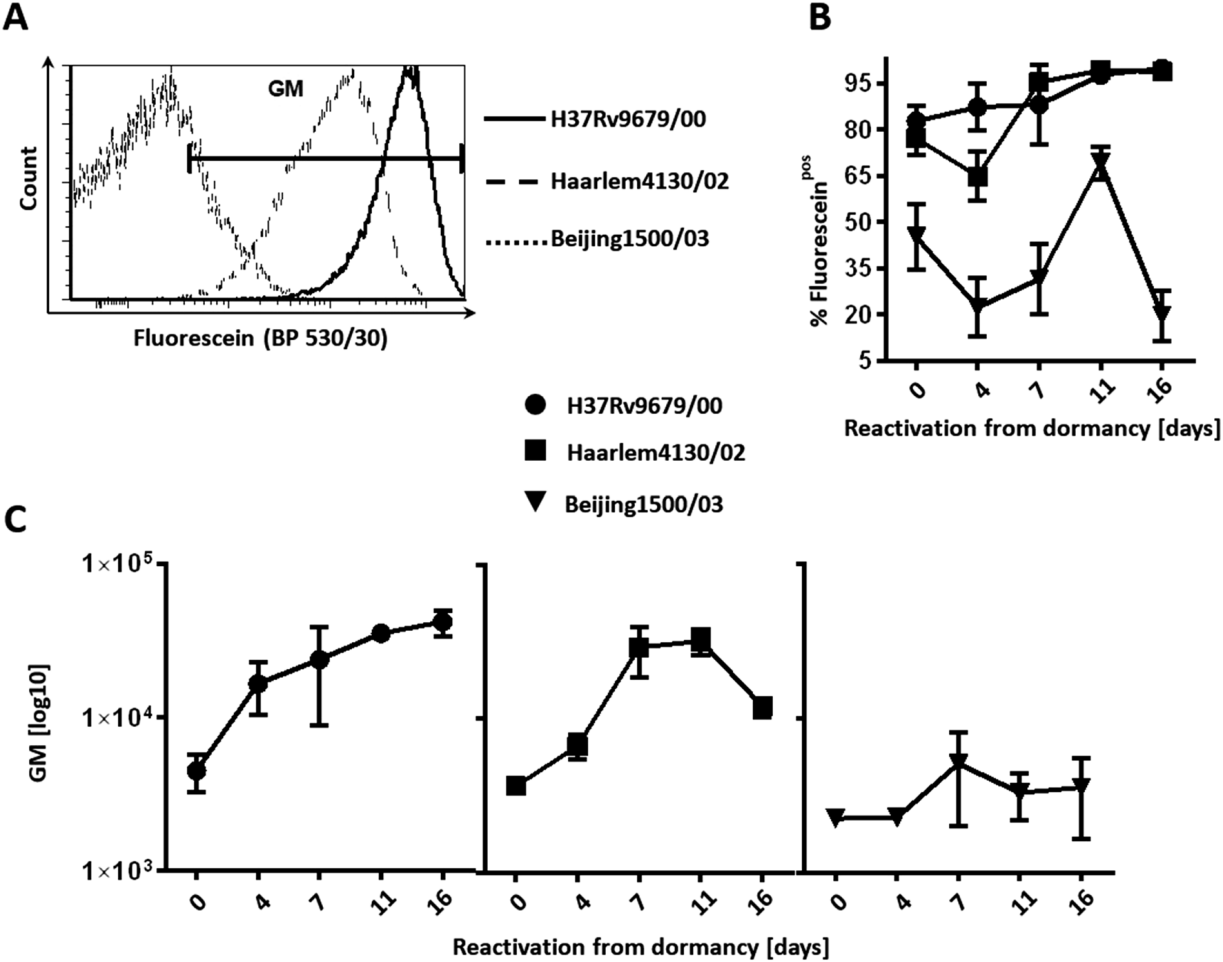
H37Rv (L4) and Haarlem (L4), but not Beijing (L2) recovered metabolic activity after reactivation from dormancy. Anaerobic mycobacterial strains from d35 of hypoxia-induced dormancy were inoculated in fresh medium and incubated in aerated conditions at 37 °C with FDA for 20 min. Example of a histogram of fluorescein-positive mycobacteria at d16 after reactivation after incubation at 37 °C with FDA for 20 min (**A**). Per cent of fluorescein-positive cells (**B**) and geometric mean (GM) fluorescence intensity (**C**) of mycobacterial strains within gate GM were measured at indicated time points. One representative experiment (triplicates) out of two is shown.

Dormant but still viable bacteria can regain cultivability and restore metabolic activity and growth, when favorable conditions are restored. We initiated reactivation of late dormant cultures (35 days of oxygen starvation) by inoculating them in fresh medium and incubating under aerobic conditions. While H37Rv (L4) 9679/00 and Haarlem (L4) 4130/02 showed similar high reactivation rates, no restoration of growth was observed for the Beijing (L2) clinical isolate 1500/03 (Fig. 3). Moreover, we performed additional experiments with other clinical isolates Beijing (L2) 12594/02, Beijing (L2) 49/02, and Haarlem (L4) 2336/02 (Supplementary Fig. S5). None of the Beijing (L2) strains reactivated in the conditions tested, while Haarlem (L4) 2336/02 did. In this context, we assessed the metabolic activity of the MTBC strains by hydrolysis of FDA via flow cytometry at different time points after reactivation from 35 days of oxygen starvation (Fig. 4). Quickly after oxygen exposure, Euro-American (L4) strains (H37Rv, Haarlem) exhibited increased mean fluorescence intensities after FDA addition, indicating recovery of metabolic activity (Fig. 4A,C). In contrast, mean fluorescence intensities of Beijing (L2) were still low. Also, percentages of mycobacterial cells considered to be positive for fluorescein were relatively high directly after resuscitation and even increasing over the time course of several days in case of Euro-American (L4) clinical isolates, while percentages of fluorescein-positive Beijing (L2)isolates remained low (Fig. 4A,B). Again, this was accompanied by a shift towards higher mean fluorescence intensities over time (H37Rv (L4), Haarlem (L4)) compared to Beijing (L2), which intensities remained at relatively low levels (Fig. 4C) indicating impaired metabolic activity.

**Figure 5:**
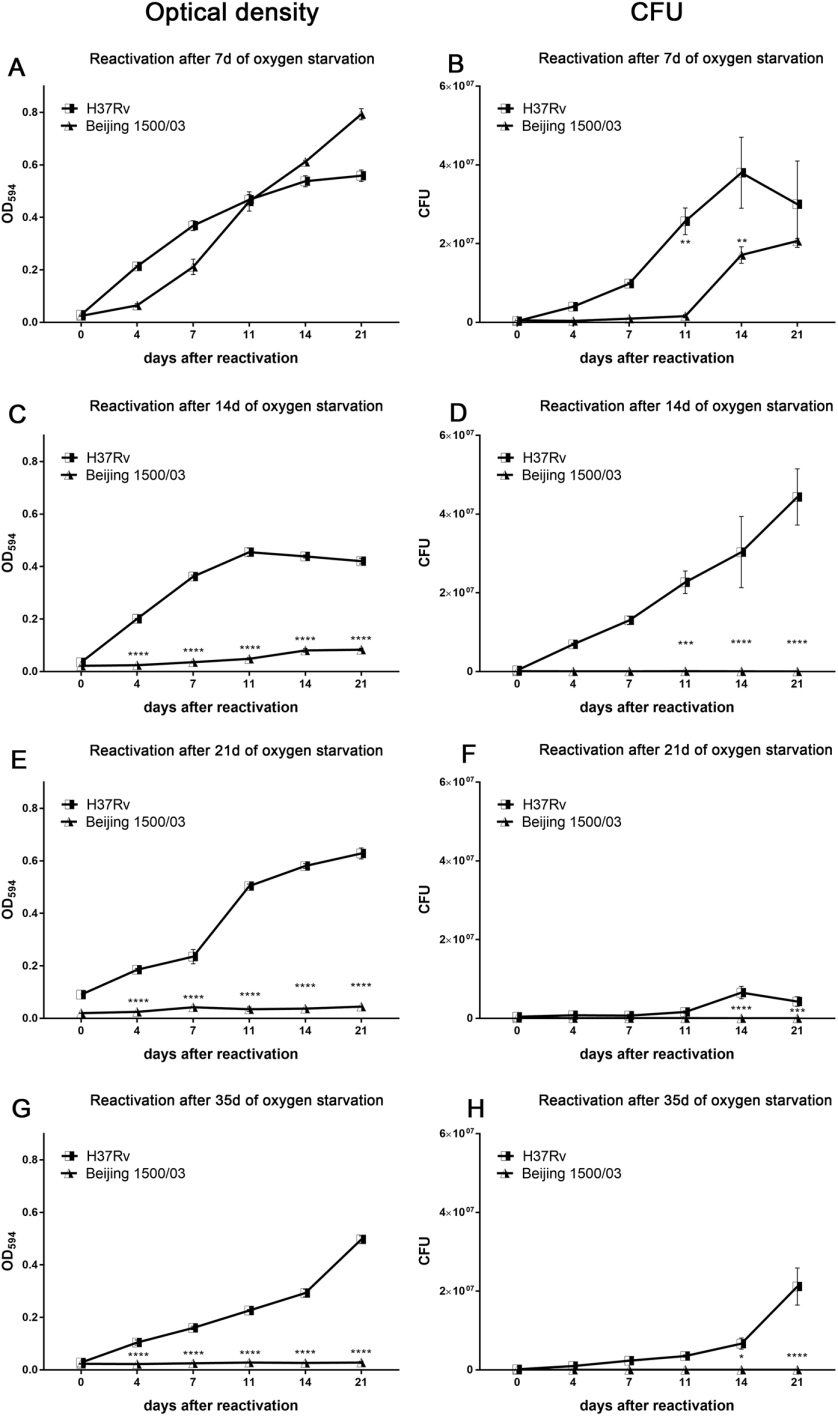
Beijing (L2) reactivated only after a short, but not after prolonged periods of O_2_ starvation. After O_2_ starvation for indicated time periods, cultures were inoculated (1:10) in fresh medium at indicated time points and growth recovery was followed by OD_594_ (**A**,**C**,**E**,**G**) or CFU (**B**,**D**,**F**,**H**). Beijing (L2) 1500/03 did not recover from O_2_ starvation for 14, 21 and 35 days, but did recover from O_2_ starvation for 7 days. In contrast, H37Rv (L4) reactivated after all O_2_ starvation periods. n = 1 independent experiments with triplicates in each. *p < 0.05, **p < 0.005, ***p < 0.005, ****p < 0.0001, two-way ANOVA.

To provide further evidence of the impaired survival observed in Beijing (L2) strains and to better define at which stage of starvation the bacteria were losing viability, we performed time-course experiments, in which the MTBC strains were reactivated by inoculation in fresh medium after 7, 14, 21, or 35 days of O_2_ starvation (Fig. 5). H37Rv (L4) 9679/00 and Beijing (L2) strain 1500/03 regained growth after 7 days of oxygen starvation (Fig. 5A,B). However, in contrast to H37Rv (L4) 9679/00, Beijing (L2) strain 1500/03 showed impaired ability to recover from hypoxia after 14, 21 and 35 days (Fig. 5C–H). H37Rv (L4) 9679/00 regained growth after all investigated periods of oxygen starvation up to 35 days as confirmed by OD measurement and CFU assay. Similarly, Haarlem 4130/02 (L4) also successfully resuscitated after 7 days and 26 days of oxygen starvation (Supplementary Fig. S6).

**Figure 6:**
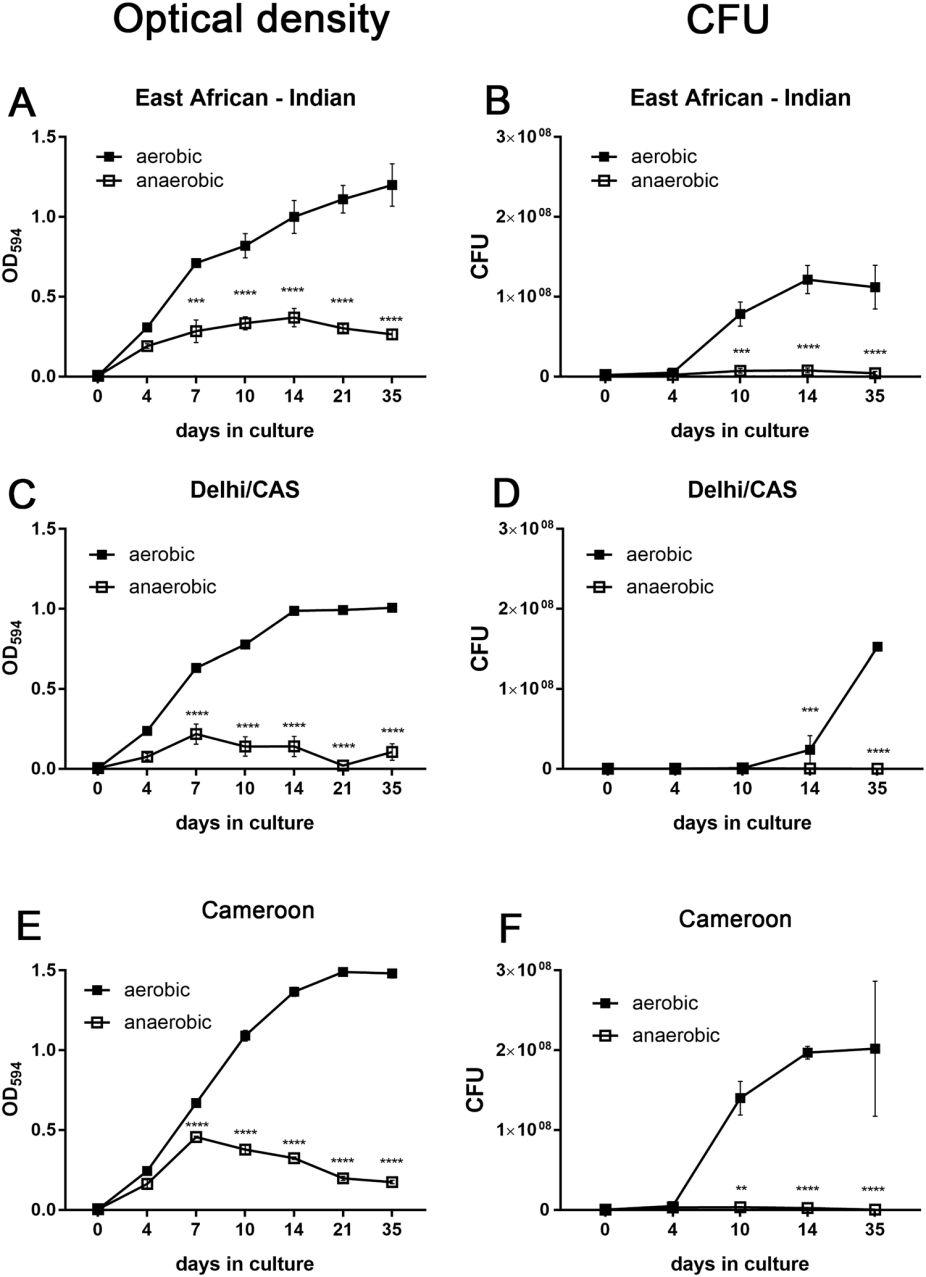
O_2_ depletion induced dormancy in EAI (L1), Delhi/CAS (L3), and Cameroon (L4). Approximately 2 × 10^6^ (OD = 0.004) mycobacteria were cultured with or without O_2_ depletion. At indicated time points, OD was measured (**A**,**C**,**E**) or cultures were plated for CFU assay (**B**,**D**,**F**). O_2_ depletion led to significantly reduced bacterial growth for all strains at indicated time points. *p < 0.05, **p < 0.005, ***p < 0.005, ****p < 0.0001, two-way ANOVA.

**Figure 7:**
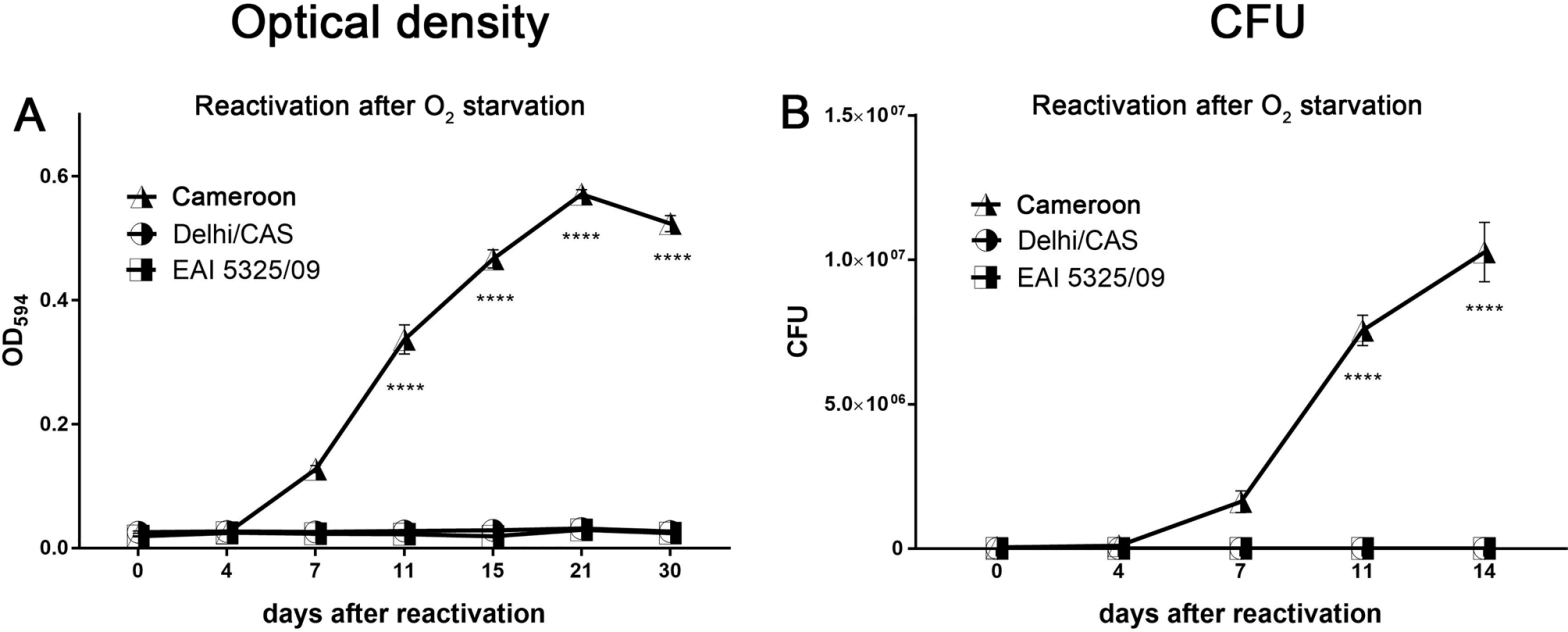
After hypoxia-induced dormancy, Cameroon (L4), but not EAI (L1) and Delhi/CAS (L3) reactivated after oxygen exposure. 35 days after oxygen starvation, *M. tuberculosis* strains were inoculated in fresh medium under atmospheric oxygen conditions. At indicated time points, mycobacterial growth was measured by optical density (**A**) or bacteria were plated for CFU analysis (**B**). Growth curves of Cameroon (L4) were significantly higher compared to EAI (L1) and Delhi/CAS (L3) from day 11 on. n = 3 independent experiments (EAI, Cameroun), n = 1 independent experiment (Delhi/CAS) with triplicates in each, ****p < 0.0001, two-way ANOVA.

To further extend our investigation to a wider panel of MTBC strains, we selected additional strains from other (sub-)lineages, namely a Cameroon (L4) strain (5390/02), an EAI (L1) strain (5325/09), and a Delhi/CAS (L3) strain (8538/03). Upon oxygen starvation, all strains acquired a dormant state as revealed by strongly reduced growth curves assessed by OD measurement (Fig. 6A,C,E) and CFU analysis (Fig. 6B,D,F). Growth curves under aerobic and anaerobic conditions were comparable between the different strains. Importantly, upon resuscitation after 35 days of oxygen depletion, only the Cameroun (L4) strain successfully reactivated (Fig. 7) and proliferated again. EAI (L1) and Delhi/CAS strains did not recover from dormancy and CFU analysis showed no live bacteria at all (Fig. 7b).

In further experiments, two other EAI (L1) clinical isolates were also not able to recover from an oxygen depletion period of 35 days (Supplementary Fig. S7). Interestingly, time course experiments revealed that the EAI (L1) isolate 1979/03 successfully regained proliferation after a short period of dormancy (7 days), but not after prolonged periods (Supplementary Fig. S7), a time kinetic very similar to that of the Beijing (L2) clinical isolate 1500/03 (Fig. 5).

Taken together, all MTBC strains tested in this study entered a dormant state upon oxygen starvation as manifested in growth reduction or inhibition and decreased or arrested metabolism. Importantly, strains belonging to the Euro-American lineage (L4), namely H37Rv, Haarlem and Cameroun, successfully reactivated even after prolonged periods of oxygen depletion when exposed to atmospheric oxygen. In contrast, strains that do not belong to the Euro-American lineage, namely Beijing (L2), EAI (L1) and Delhi/CAS (L3), did not resuscitate from prolonged O_2_ starvation and no viable mycobacteria were detectable anymore as monitored by CFU analysis.

### Transcriptome analysis revealed a distinct expression response of Beijing (L2) vs. H37Rv (L4) upon oxygen depletion

To gain insights into gene regulations upon dormancy entry by oxygen depletion, we performed transcriptome analysis for the exemplary strains H37Rv (L4) and Beijing (L2) to investigate differential gene expression patterns.

First, we determined a reasonable timeframe for microarray analysis in which strains that were not able to resuscitate from prolonged oxygen depletion entered a hypoxia-induced dormant state but did not die, yet. Thus, high mRNA yield and quality could be collected. At this time point, most profound differences in expression profiles between oxygenated and oxygen-starved cultures have been observed. This phase corresponded to the stationary NRP2 phase, also reflected by the growth profiles (see Fig. 1). Upon hypoxia-induced dormancy, we observed a strong upregulation of stress- and oxygen starvation-related genes by H37Rv (L4), commonly referred to as the DosR regulon, confirming previously described findings (Supplementary Table S1, Supplementary Fig S8b)^26,27^. Analysis of dormant vs. oxygenated H37Rv (L4) cultures revealed 471 upregulated and 483 downregulated genes with a fold change of at least 2, including 36 induced (out of 48) DosR regulon genes (Supplementary Table S1, Supplementary Fig. S8b). Increasing the cut-off to an at least four-fold expression change reduced the number of up- and down-regulated genes to 93 and 335, respectively, still including 32 of the 48 genes that have been previously associated with dormancy^26^. Therefore, the NRP-2 stage after 10 days of O_2_ starvation was chosen for comparative transcriptome analyses of Beijing (L2) and H37Rv (L4).

**Figure 8:**
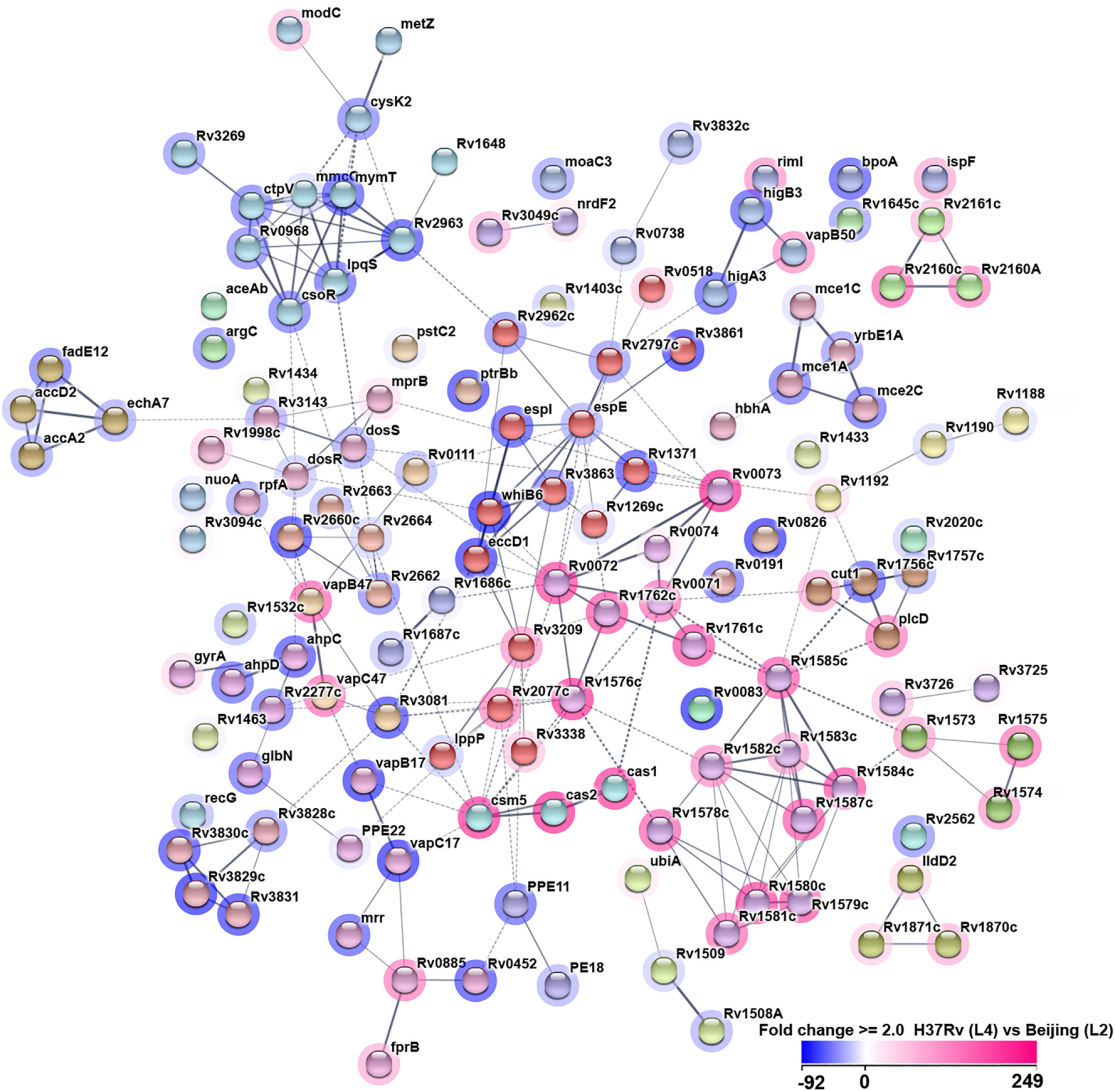
STRING association network of differentially expressed genes. Functional association and interactions for the 139 ≥ two-fold differentially expressed genes at day 10 between H37Rv (L4) and Beijing (L2) (three independent experiments per condition). Links between genes show their joint contribution to specific biological functions and the degree of confidence prediction is represented by the thickness of the line. Lines are either dashed or solid if genes are found to be clustered by the cluster algorithm MCL run on the distance matrix obtained from the STRING global scores. Nodes are colored by clusters and every color represents a different cluster. Halos around nodes show the degree of up- or down-regulation in shades of blue and pink, respectively, in H37Rv (L4) compared to Beijing (L2).

With 86 repressed and 53 induced genes in H37Rv (L4) compared to Beijing (L2) at d10 of hypoxia-induced dormancy entry, we observed distinct expression patterns for each strain (Supplementary Table S2, Supplementary Fig. S8a). The STRING functional network analysis revealed with 228 edges between the 139 nodes of differentially expressed genes significantly more interactions than expected (81, enrichment p-value < 1 × 10^16^, Fig. 8).

Here, we aim to only provide a general expression pattern overview upon dormancy induced by oxygen starvation of the tested Beijing (L2) strain as an exemplary non-Euro American strain. We refrain from describing single genes in detail since no further functional analyses have been conducted, e.g. confirmation by RT-qPCR or gene knock-outs. However, we found an individual expression pattern for the Beijing (L2) strain compared to H37Rv (L4).

## Discussion

The results of this study reveal that reactivation after dormancy induced by oxygen depletion was a specific characteristic only of those MTBC clinical isolates that belong to the Euro-American lineage (L4) and not a general feature of all branches of the MTBC (an overview of all strains investigated in this study is shown in Supplementary Table S3). Thus, our findings suggest that survival of hypoxia-induced dormancy is not a general feature of all MTBC strains as claimed before. This observation may contribute to our understanding of the pathobiology of one of the most successful bacterial pathogens worldwide regarding infection and spread.

Although a broader spectrum of other clinical isolates has to be tested for generalized assertion, enhanced survival after oxygen starvation might be a key feature of Euro-American strains (L4) contributing to the global success of strains of this lineage that are virtually found in all parts of the world as shown in our recent investigation^6^. While pathobiological differences among, e.g. Euro-American (L4), Beijing (L2), and EAI (L1) strains have been previously shown in experimental models^12^, it is tempting to speculate that improved survival after oxygen starvation, for instance within granulomas, might have contributed to the global distribution of Euro-American (L4) strains.

With regard to its proposed clinical importance, adaptation to oxygen-limiting conditions is among the most widely studied mycobacterial stress condition that defines latent infection state^18^. Limited *M. tuberculosis* replication and long-term survival during hypoxic starvation in vitro likely represent the mycobacterial state within granulomas. However, previous hypoxia studies mainly focused on the reference strain H37Rv (L4). Still, it is assumed that survival of hypoxia-induced dormancy is a general feature of all MTBC strains^18^. Our investigation of a well characterized set of clinical isolates belonging to Euro-American (L4) and non-Euro-American (L1, L2, L3) lineages in the Wayne model showed that this is not the case. On the contrary, the results obtained provide evidence that the ability of clinical MTBC strains to survive long-term anaerobic conditions was restricted exclusively to strains belonging to the Euro-American lineage (L4) with respect to the strain collection of this study. While growth rates upon hypoxia-induced dormancy were similar among all strains investigated, the ability to resuscitate was only observed for clinical isolates belonging to the Euro-American (L4) lineage regarding recovery of proliferation and metabolic activity. Time course experiments revealed that Beijing (L2) strains were able to survive only relatively short periods of self-generated O_**2**_ starvation, representing the microaerobic NRP-1 phase. In contrast, Euro-American (L4) strains resuscitated also after prolonged periods of complete O_2_ depletion, representing the anaerobic NRP-2 phase. It has been shown before that anaerobic conditions exist in granulomas and at sites of infection in the human host as well as in animal models that develop necrotizing granulomas, such as guinea pigs and non-human primates^21^. Moreover, O_2_ depletion drives the effectiveness of granulomas to restrict *M. tuberculosis* infection in a computational biology study^28^. These findings have been further supported by the observation that Metronidazole, a drug that is only mycobactericidal under strict anaerobic conditions, is efficient in anti-Tb treatment in humans, macaques and guinea pigs^21,29,30^. To explain the fact of successful spreading of EAI (L1), Beijing (L2), and Delhi/CAS (L3) clinical isolates, our data suggest that infection dynamics between the used clinical isolates belonging to the Euro-American linage (L4) and those belonging to the non-Euro-American linages (L1–3) differ in a way that the latter benefit from reduced periods of latent infection and avoiding anaerobic conditions by causing disease more rigorous and quicker. Indeed, in a cohort study in The Gambia, de Jong and colleagues showed that tuberculosis household contacts that were infected with the Beijing (L2) strain most likely progressed to disease as compared to other linages and *M. africanum* (L5, L6)^11^. Importantly, infection rates did not differ.

Microarray data collected at the transition from NRP-1 to NRP-2 phase of dormancy confirmed a distinct transcriptional profile for Beijing (L2) strains. The STRING interaction network showed with 228 edges a significant enrichment. This means that there are more interactions among themselves than what would be expected for a random set of similar size drawn from the genome. This enrichment indicates that entities are at least partially biologically connected as a group. As published before, we could also show that several genes belonging to the DosR regulon were induced in H37Rv (L4) upon hypoxia-induced dormancy entry^27^. Thus, our transcriptomic data is in line with previous publications, validating our microarray results. Of note, in this study, we only provide a general transcriptomic overview of several clustered gene sets obtained by microarray assays. Oxygen starvation survival has been attributed to upregulation of genes belonging to DosR in H37Rv^31^. Interestingly, other studies showed that Beijing (L2) constitutively over-express the complete DosR regulon including both regulators *dosR* and *dosS*^32–34^. However, Beijing (L2) strains did not survive prolonged hypoxic stress periods in our study. A deeper characterization of these transcriptomic network functions of single regulated genes is required to further elucidate their role in mechanisms of dormancy and reactivation of disease.

In summary, our results suggest that dormancy survival of clinical MTBC isolates is lineage-dependent. The ability to recover from prolonged periods of O_2_ starvation was exclusively observed in Euro-American (L4) strains but not in other tested strains. We demonstrate that the used Euro-American (L4) strains were more prone to adapt to hypoxic stress environments, to enter a state of dormancy and to recover viability after prolonged anaerobiosis. These findings question the claim that resuscitation from dormancy after oxygen starvation is a general feature of all MTBC strains. This observation could prove important for the development of host-directed therapies and drugs that target the dormant state.

## Material and methods

### Cultures and strains

Frozen aliquots of mycobacteria were thawed at room temperature and passed through a syringe for single cell separation. 200 µL bacterial culture was added to 5 mL Middlebrook 7H9 broth (Difco, Detroit, MI, USA) containing 0.2% glycerol, 0.05% Tween 80, and 10% oleic acid-albumin-dextrose-catalase (OADC) growth enrichment (Becton Dickinson, Cockeysville, MD, USA). Cultures were incubated at 37 °C until they reached an OD of ~ 0.2. Two mL pre-culture (at OD = 0.2) were added to 15–18 mL Dubos medium (Becton, Dickinson) supplemented with 0.05% Tween 80 and 10% OADC and incubated in roller bottles (Corning) at 37 °C for 7–10 days. At an OD of 0.4–0.5 (log phase) the cultures were diluted to OD = 0.004 in Dubos complete medium and used for dormancy experiments. Colony forming unit (CFU) analyses were calculated by serial dilutions plated on Middlebrook 7H11 agar containing 0.5% glycerol and 10% OADC growth enrichment. All CFU values in this study represent CFU/mL culture. A list of all used clinical isolates can be found in Supplementary Table S3.

### Dormancy experiments

Experiments were performed as previously described by Wayne et al. Briefly, screw-capped glass tubes (Max Wiegand, Germany, total volume: 18.5 mL) have been filled with 10 mL mycobacterial culture (OD = 0.004; Head Space Ratio (HSR): 0.54), tightly closed, wrapped with Parafilm and incubated at 37 °C on a magnetic stirring plate (Variomag, Thermo Scientific) at 130 rpms 7–35 days. During oxygen starvation, OD_594_ was measured with a portable photometer (pHotoFlex STD, WTW) without opening the tubes. Oxygen consumption was monitored via decolorization of the redox indicator methylene blue (1.5 µg/mL) in control tubes. Methylene blue served as a visual indicator of hypoxia. Loss of blue color occurs after 10–17 days, depending on the strain. For aerated controls, loosely capped glass tubes with magnetic stirrer or square bottles were filled with 7 mL culture and incubated at 37 °C. For reactivation experiment, dormant cultures (35 days of oxygen starvation) were inoculated in fresh Dubos medium (supplemented with 10% OADC and 0.05% Tween 80) at indicated time points and incubated in aerated conditions at 37 °C.

### Flow cytometric assay

Flow cytometric tests of metabolically active mycobacteria using fluorescein diacetate (FDA, Sigma-Aldrich) were conducted as described before^35,36^. Mycobacteria hydrolyze FDA to free fluorescein via non-specific cellular esterases. Accumulation of free fluorescein in metabolically active mycobacteria was quantified. FDA was dissolved in DMSO (5 mg/mL) and diluted to a concentration of 500 ng/mL in PBS (pH 7.4). OD of mycobacterial cultures were diluted to a final value of 0.04 within test samples (400 µL/test). Mycobacterial cultures (OD = 0.04) were incubated in PBS (controls) or in PBS plus FDA (FDA = 250 ng within 400 µL/test) at 37 °C for 20 min. Background fluorescence was subtracted by measurement directly after addition of FDA without incubation time. A minimum of at least 15,000 events (doublets excluded) were measured and mycobacteria were analyzed using a Facs Canto II flow cytometer (BD Bioscience). Gating strategy is depicted in supplementary Fig. S9. Free fluorescein within the mycobacteria was measured using a bandpass filter (530/30) after 488 nm excitation. Data analysis was performed utilizing FCS Express 5 (De Novo Software).

### RNA extraction and transcriptome analysis

Total RNA was extracted from dormant cultures 0 and 10 days after oxygen depletion and labeled and hybridized with customized microarrays. Bacteria were inactivated with 1:1 (v/v) guanidine thiocyanate buffer (300 g guanidin thiocyanate, 2.5 g N´laurylsarcosine, 3.7 g sodium citrate in 500 ml Diethylpyrocarbonate (DEPC)-H_2_O, 3.5 mL ß-Mercaptoethanol), centrifuged at 3 k×*g* for 30 min, resuspended in lysis binding buffer from MirVana kit (Thermo Fisher Scientific), and disrupted in lysing matrix B beads (MP Biomedicals) in a FastPrep shaker (FastPrep-24, MP Biomedicals). RNA isolation was performed according to MirVana kit manufacturer’s instructions. RNA samples were stored at − 20 °C. For microarray experiments, 50 ng of total RNA from three independent experiments for each condition were labeled using the “Low Input Quick AMP WT Labeling Kit, one color” (Agilent Technologies, Santa Clara, USA) according to the manufacturer`s instructions. Briefly, mRNA was amplified by reverse transcription. cDNA was transcribed by T7 RNA Polymerase integrating Cyanine 3 dye (Cy3)-labeled Cytidintriphosphate (CTP). Purified, labeled RNA (600 ng) was hybridized to custom Agilent 8 × 15 K microarrays (OakLabs), eArray design ID 064078, based on H37Rv NC_000962 complemented by probe sets of four other MTBC lineage strains. For this analysis only the RV gene features from H37Rv were evaluated. Microarrays were analyzed by Agilent GeneSpring 13.1.1 software with percentile shift normalization to the 75th percentile. Expression was analyzed in the biological significance workflow in gene level experiments and differentially expressed genes were selected using the moderated t-test, a corrected p-value of ≥ 0.05 (FDR, Benjamini-Hochberg), and a fold change ≥ 2. Hierarchical clustering of strains was performed using Euclidian distance and Ward’s linkage rule. A functional association network was built with STRING v11 as described before^37^ using only the differential expressed genes as query and showing interactors. Links between genes were set to show the confidence of predicted interaction. MCL clustering was initialized from within the STRING application with an inflation parameter of 1.8 for optimized accuracy and separation as shown before^38^. Normalized expression and raw microarray data are available at NCBI GEO (https://www.ncbi.nlm.nih.gov/geo/) under the accession numbers GSE159982 and GSE159983.

### Statistical data analysis

Each graph represents means with error bars indicating the SD. n-values in figure legends represent replications of independent experiments, each including triplicates. All data of individual experiments have been pooled and depicted in one graph. Statistical significances are indicated in the figures (*, p < 0.05; **, p < 0.01; ***, p < 0.001; ****, p < 0.0001) and mentioned in the figure legends. Differences were considered statistically significant when p < 0.05. Statistical tests used are indicated in the figure legends and were calculated with Graph Pad Prism 7.

## Supplementary Information

Supplementary Information 1.

Supplementary Information 2.

Supplementary Information 3.

Supplementary Information 4.

Supplementary Information 5.

## Data Availability

Data are available upon request.
