# Supplementary material for: Survival of hypoxia-induced dormancy is not a common feature of all strains of the Mycobacterium tuberculosis complex
Source: Sci Rep. 2021 Jan 29;11:2628. doi: 10.1038/s41598-021-81223-6 (PMC7846770; doi:10.1038/s41598-021-81223-6)
Supplement: Supplementary file 3 — Supplementary Information 3. [file 41598_2021_81223_MOESM3_ESM.pdf]

Survival of hypoxia-induced dormancy is not a common feature of all strains of the *Mycobacterium tuberculosis* complex

Barbara Tizzano[1,+], Tobias K. Dallenga[2,+,*], Christian Utpatel[1,+], Jochen Behrends[3], Susanne Homolka[1], Thomas A. Kohl[1], and Stefan Niemann[1,4,*]

[1]Molecular and Experimental Mycobacteriology, Research Center Borstel, Leibniz Lung Center, Parkallee 1-40, 23845 Borstel, Germany

[2]Cellular Microbiology, Research Center Borstel, Leibniz Lung Center, Parkallee 1-40, 23845 Borstel, Germany

[3]Core Facility Fluorescence Cytometry, Research Center Borstel, Leibniz Lung Center, Parkallee 1-40, 23845 Borstel, Germany

[4]German Center for Infection Research, Borstel Site, Borstel, Germany

[+]These first authors contributed equally to the article

* Corresponding authors: sniemann@fz-borstel.de, phone: +49 4537 188 7620, Address: Research Center Borstel, Leibniz Lung Center, Parkallee 1, 23845 Borstel, Germany

tdallenga@fz-borstel.de, phone +49 4537 188 5561, Address: Research Center Borstel, Leibniz Lung Center, Parkallee 29, 23845 Borstel, Germany

Supplementary figures

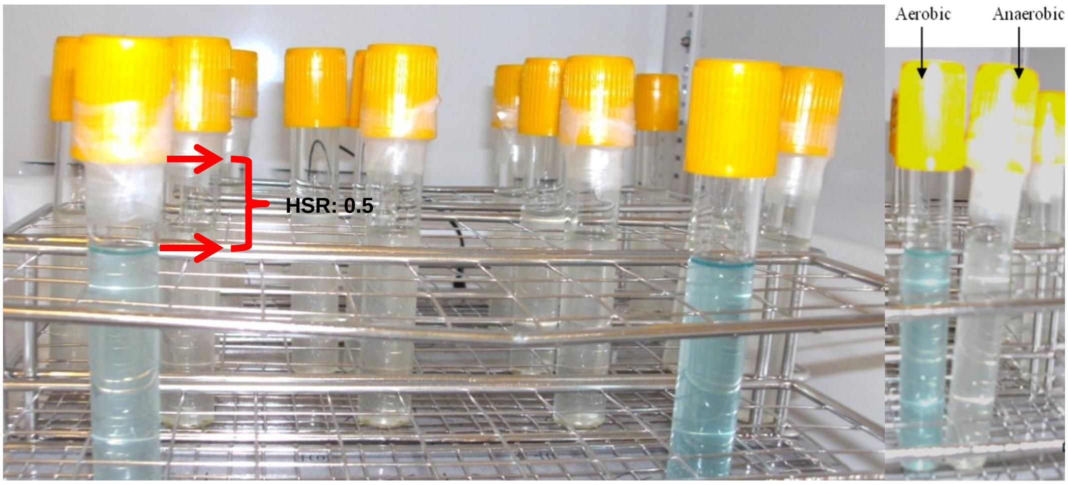

Figure S1: Wayne model system. *M. tuberculosis* strains were incubated in anaerobic conditions in Dubos/OADC/Tween80 at 37°C and stirring at 130 rpms. Methylene blue fading was used as indicator of oxygen depletion. Cultures in tightly closed tubes became colorless within 14-17 days. Cultures in loosely capped tubes remain blue. The head–space ratio (HSR) was 0.5.

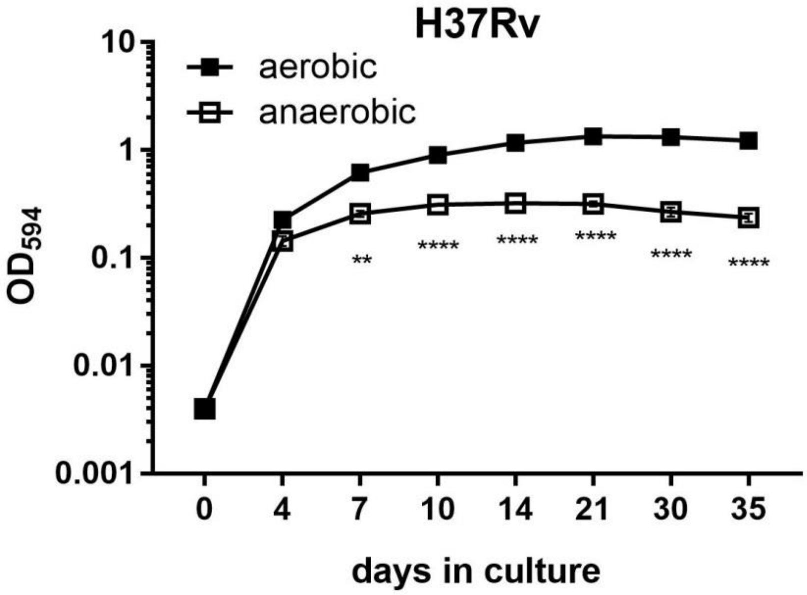

Figure S2: O2 depletion-induced dormancy in H37Rv (L4) depicted in log-scale according to the Wayne model. Approximately 2 x 10^6 (OD = 0.004) mycobacteria were cultured with or without O2 depletion. At indicated time points, OD was measured. O2 depletion led to reduced bacterial growth. n = 1 with triplicates, **** = $p < 0.0001$, ** = $p < 0.005$, two-way ANOVA

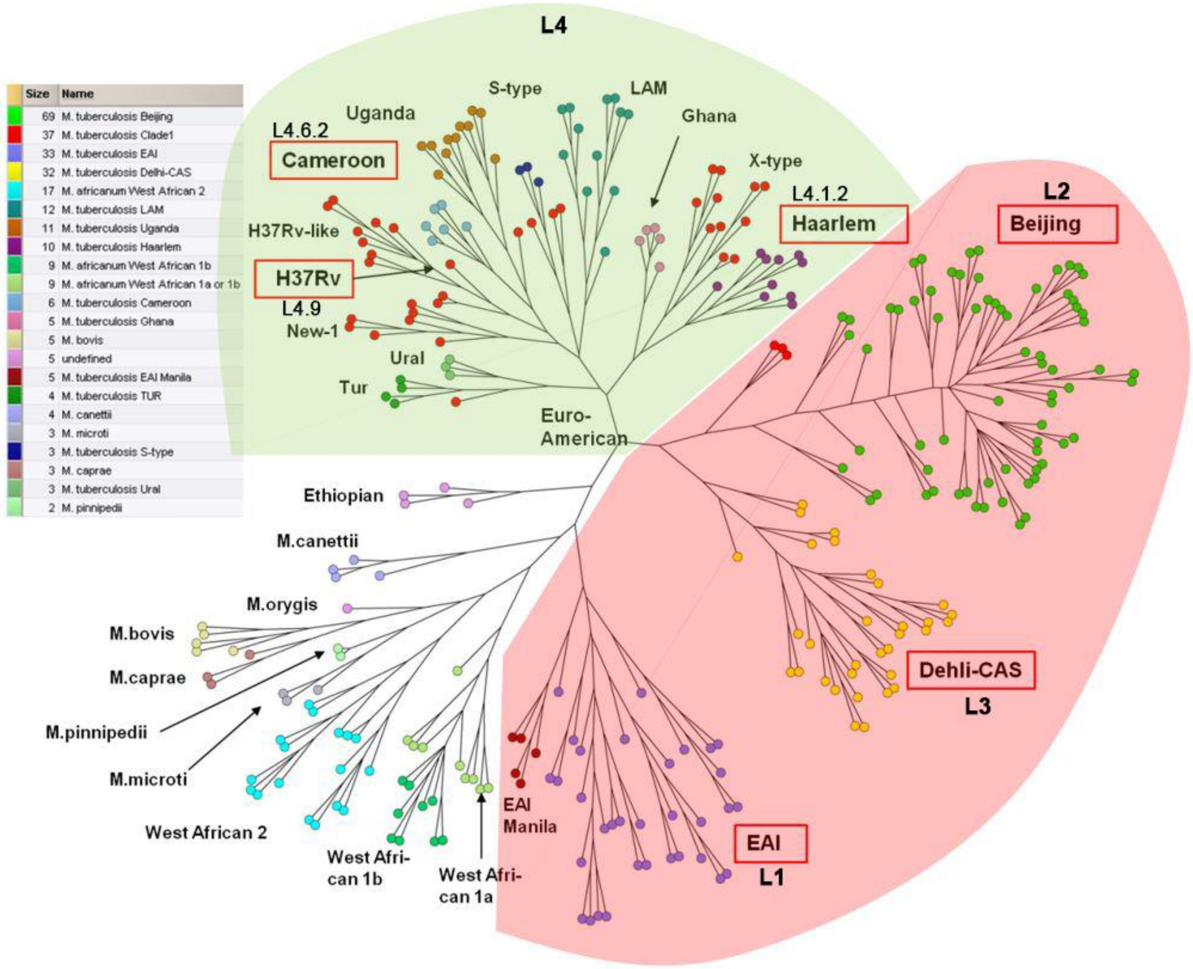

Figure S3: Phylogenetic tree representing the diversity of the MTBC, constructed with the neighbor joining algorithm from 46,613 distinct single nucleotide polymorphism positions of 287 strains. Highlighted lineages were part of this study.

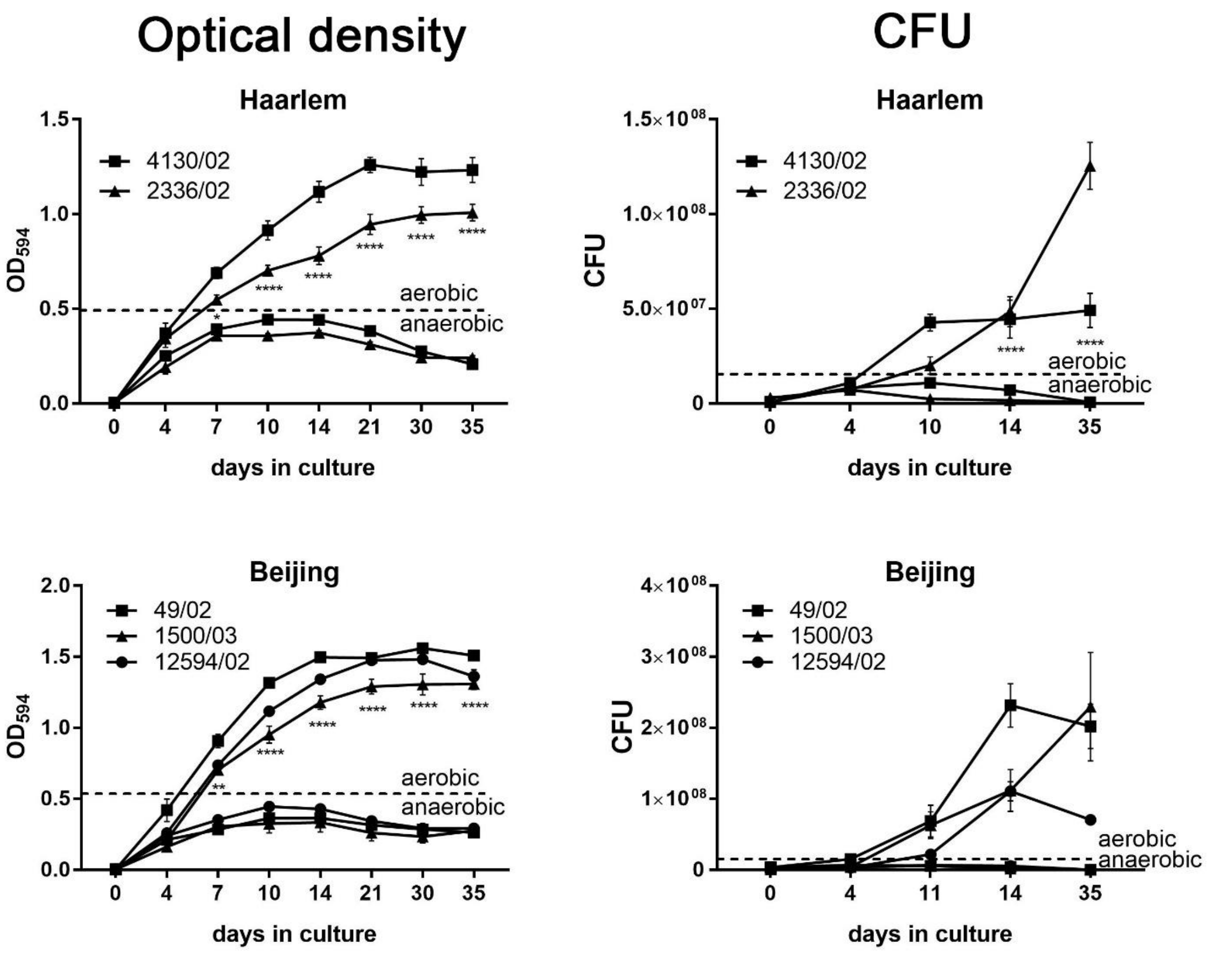

Figure S4: **O2 depletion induced dormancy also in different clinical isolates of Haarlem (L4) and Beijing (L2).** In addition to Fig. 1, also other clinical isolates of Haarlem (L4) and Beijing (L2) showed a similar dormancy phenotype. Approximately 2 x 10^6 (OD = 0.004) mycobacteria were cultured with or without O2 depletion. At indicated time points, OD was measured. O2 depletion led to reduced bacterial growth in all examined clinical isolates. n = 3 independent experiments with triplicates in each, **** = $p < 0.0001$, two-way ANOVA

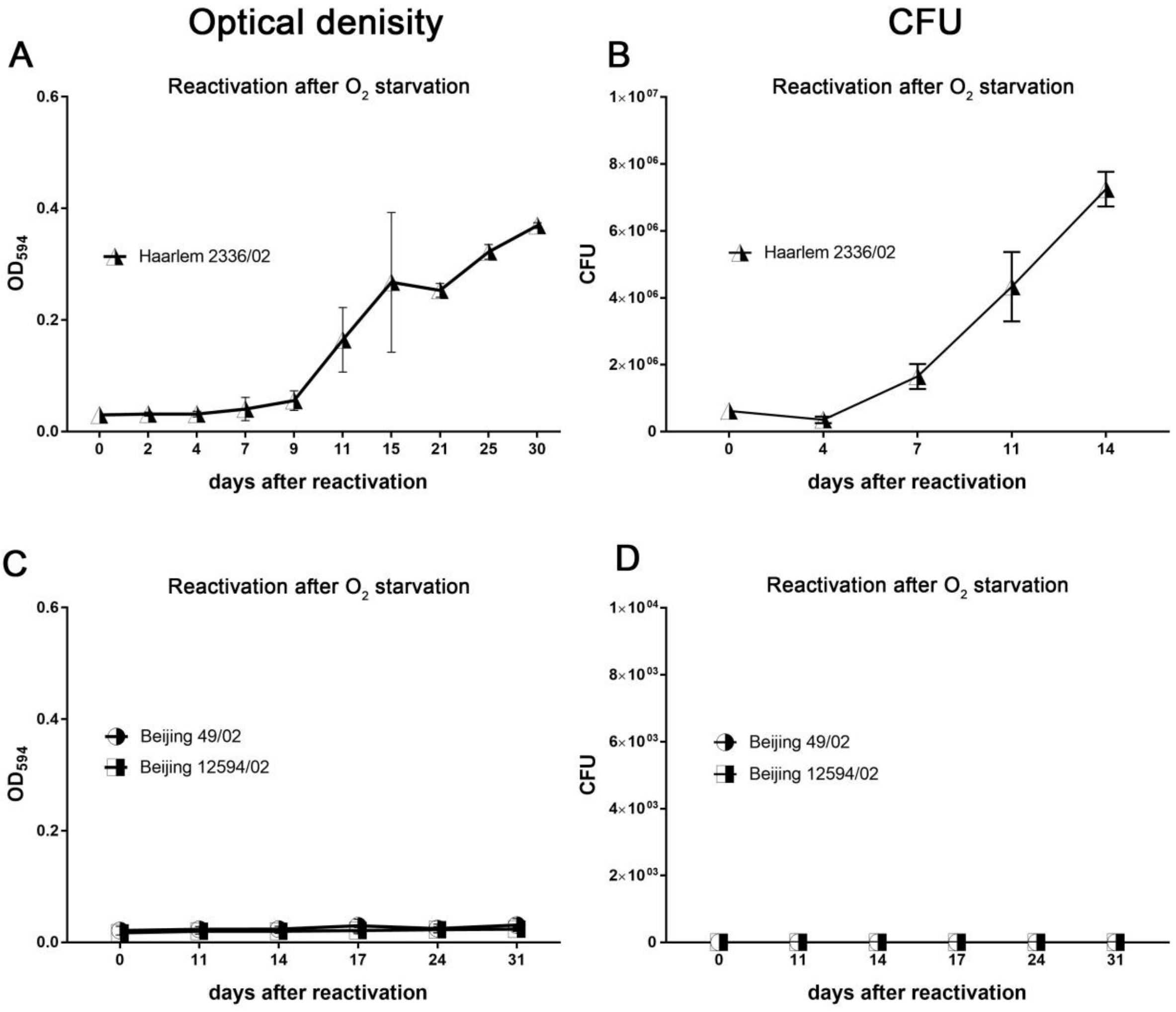

Figure S5: After hypoxia-induced dormancy, also another clinical isolate of Haarlem (L4) reactivated after oxygen exposure, but other clinical isolates of Beijing (L2) did not. In addition to Fig. 3, also other clinical isolates of Haarlem (L4) and Beijing (L2) showed similar reactivation phenotypes. 35d after oxygen starvation, *M. tuberculosis* strains were inoculated in fresh medium under atmospheric oxygen conditions. At indicated time points, mycobacterial growth was measured by optical density (A, C) or bacteria were plated for CFU analysis (B, D). n = 3 (Beijing 49/02), n = 2 (Haarlem 2336/02) or n = 1 (Beijing 12594/02) independent experiments with triplicates in each.

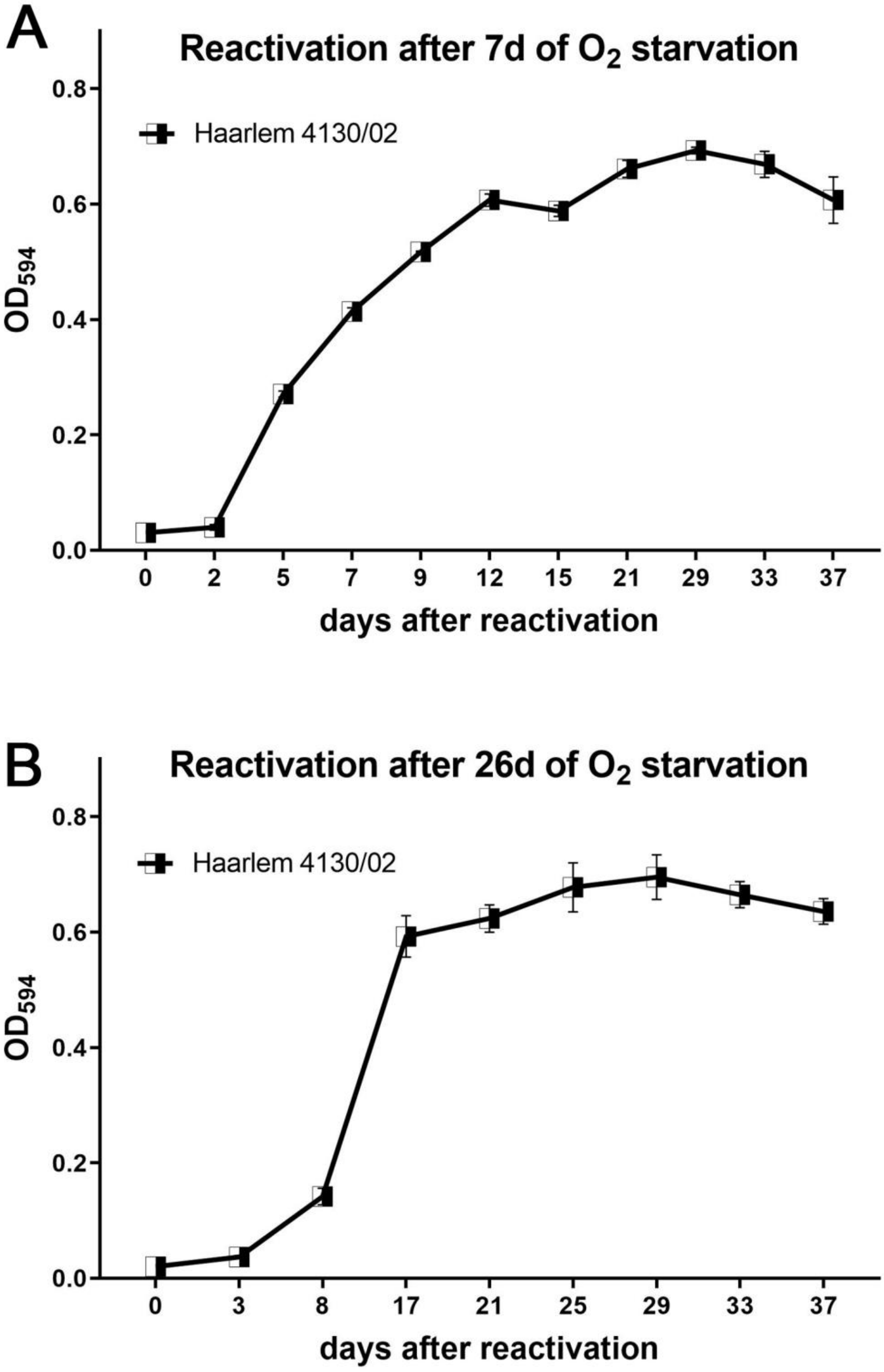

Figure S6: Haarlem 4130/02 (L4) reactivated after 7d and 26d of O_2 starvation. After O_2 starvation for 7d (A) or 26d (B), cultures were inoculated (1:10) in fresh medium at indicated time points and growth recovery was followed by OD_{594}. n = 1 independent experiments with triplicates in each.

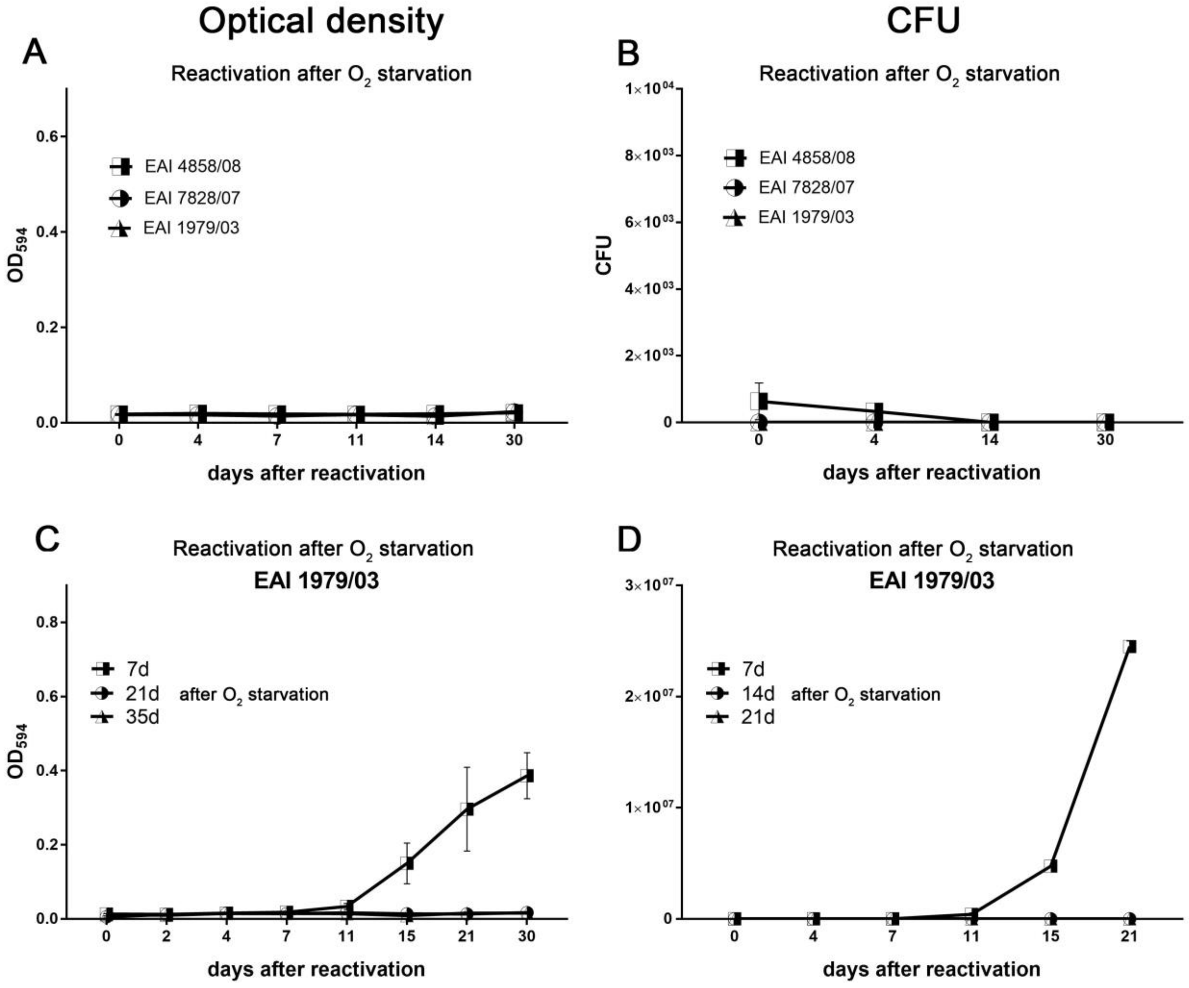

Figure S7: After hypoxia-induced dormancy, also different clinical isolates of EAI (L1) did not reactivate by oxygen exposure. In addition to Fig. 8, so other clinical isolates of EAI (L1) showed a similar phenotype after reactivation. 35d after oxygen starvation (A, B), EAI (L1) strains were inoculated in fresh medium under atmospheric oxygen conditions. At indicated time points, mycobacterial growth was measured by optical density (A) or bacteria were plated for CFU analysis (B). All clinical isolates of EAI (L1) did not recover from dormancy. Similar to Beijing (L2) in Fig. 4, EAI (L1) reactivated after relatively a short period of oxygen starvation (7d), but not after prolonged periods (21d, 35d) (C, D). n = 1 independent experiment with triplicates in each.

A

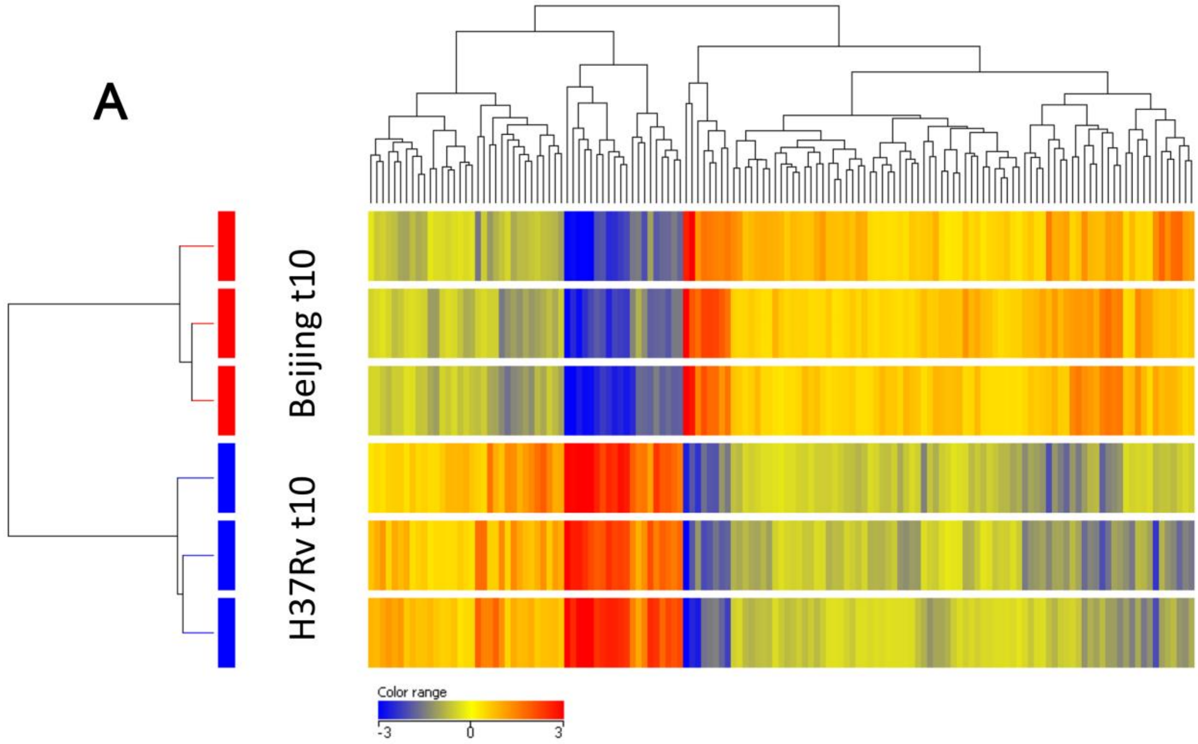

B

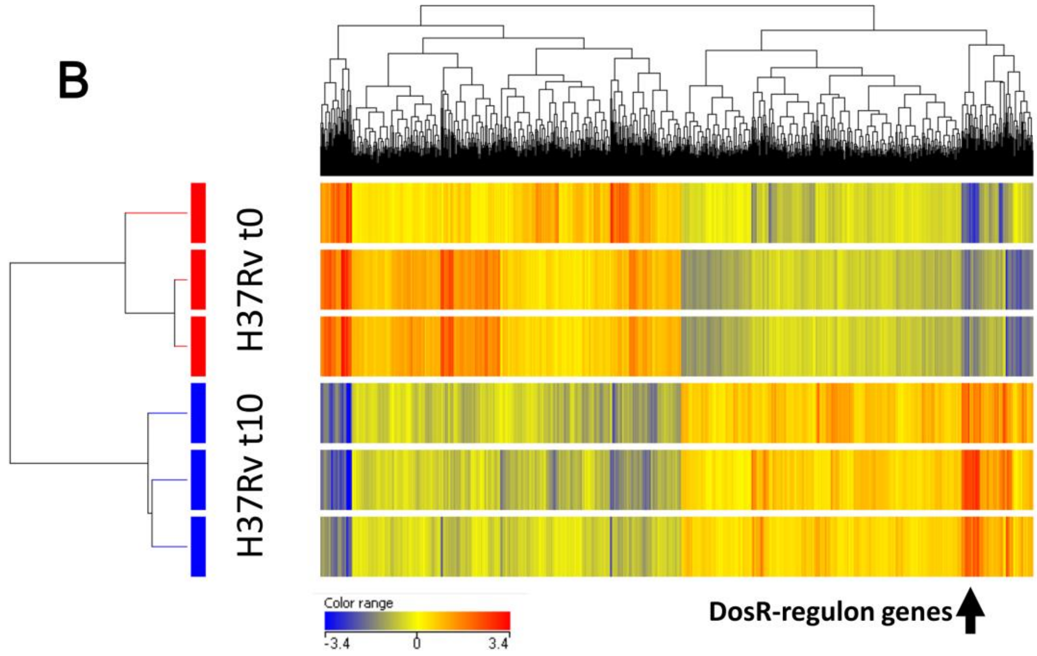

Figure S8: Hierarchical clustering of 139 differential expressed genes of H37Rv (L4) and Beijing (L2) after 10 days of oxygen depletion (A). Hierarchical clustering of 954 differential expressed genes of H37Rv (L4) from aerobic cultures and after 10 days of

oxygen depletion (B). Colored and clustered by normalized intensity values, similarity measure: Euclidian, linkage rule: Wards. n=3 independent experiments.

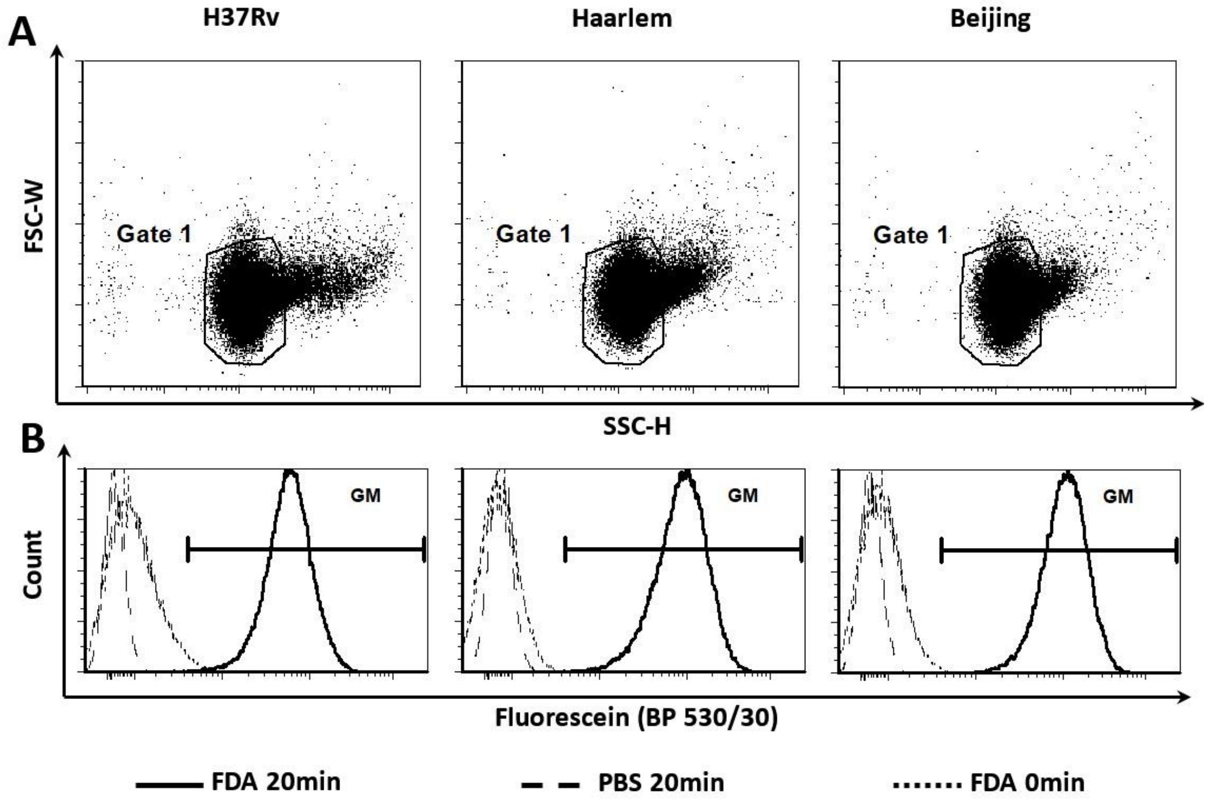

Figure S9: Exemplary gating strategy for flow cytometric analysis of FDA assays. (A) Single cell *M. tuberculosis* were selected (gate 1) by exclusion of aggregates using forward scatter width versus side scatter height. (B) Controls (dashed lines: PBS; dotted lines: FDA 0min) and FDA-positive bacteria (solid line; gate GM).

Tables

Suppl table S3 legend

Differentially expressed genes between time points (conditions) were filtered using moderated t-test, corrected p-value of ≥0.05 (FDR, Benjamini-Hochberg), and fold change ≥2. Fold change gives the absolute ratio of normalized intensities (no log scale) between average intensities of the grouped samples. Positive numbers show higher, negative numbers lower gene expression in H37Rv at day 10 compared to H37Rv at day 0.

Suppl table S2 legend

Differentially expressed genes between strains (conditions) were filtered using moderated t-test, corrected p-value of ≥0.05 (FDR, Benjamini-Hochberg), and fold change ≥2. Fold change gives the absolute ratio of normalized intensities (no log scale) between average intensities of grouped samples grouped. Positive numbers show higher, negative numbers lower gene expression in H37Rv compared to Beijing.

Lineage	Sub-lineage	Isolate	Reactivation
L4<br>Euro-<br>American	4.1.2<br>Haarlem	4130/02<br>2336/02	Yes<br>Yes
	4.6.2<br>Cameroon	5390/02<br>5400/02	Yes<br>Yes
	4.9<br>H37Rv-like	9679/00	Yes
L2<br>Beijing		49/02<br>1500/03<br>12594/02	No<br>No<br>No
L3<br>Delhi/CAS		8538/03<br>7936/01	No<br>No
L1<br>East African<br>Indian		5325/09<br>4858/08<br>7828/07<br>1979/03	No<br>No<br>No<br>No

Suppl. table S3. Summarized results regarding reactivation potential of all strains used in this study.
